# Cost-Effective and Rapid Detection of Tetrodotoxin Using Indium Tin Oxide Electrodes via In Vitro Electrophysiology and Electrochemistry

**DOI:** 10.3390/toxins17090462

**Published:** 2025-09-13

**Authors:** Naga Adithya Chandra Pandurangi, Manel M. Santafe, Angels Tudo, Nagihan Ozsoy, Fransesc X. Sureda, Mark L. Dallas, Ioanis Katakis

**Affiliations:** 1 Department of Chemical Engineering, Universitat Rovira i Virgili, 43007 Tarragona, Spain; nagaadithyachandra.pandurangi@estudiants.urv.cat; 2 Department of Basic Medical Sciences, Universitat Rovira i Virgili, C/Sant Llorenç 21, 43201 Reus, Spain; manuel.santafe@urv.cat (M.M.S.); francesc.sureda@urv.cat (F.X.S.); 3 Reading School of Pharmacy, University of Reading, Reading RG6 6UR, UK; nagihan.ozsoy@pgr.reading.ac.uk (N.O.); m.dallas@reading.ac.uk (M.L.D.)

**Keywords:** indium tin oxide (ITO), tetrodotoxin (TTX), in vitro electrophysiology, potassium chloride (KCl)-evoked, extracellular field potential (EFP), whole-cell patch clamp, Neuro-2a cells, voltage-gated sodium channels, electrochemical impedance spectroscopy (EIS)

## Abstract

The real-time, cost-effective detection of marine toxins like tetrodotoxin (TTX) remains a significant challenge for the scientific community. Traditional methods, including cell-based assays (CBAs), high-performance liquid chromatography (HPLC), and automated patch clamp (APC), are time-consuming, requiring expensive lab-based equipment and highly trained personnel. Enzyme-linked immunosorbent assays (ELISAs), lateral flow assays (LFAs), and immunosensors may not be suitable for toxin analogues. Thus, a simplified approach has been developed in this study, which involves the electrophysiological and electrochemical interrogation of N2a cells grown on ITO-coated glass electrodes by measuring extracellular field potentials (EFP) in conjunction with whole-cell patch clamp recordings and electrochemical impedance spectroscopy (EIS) measurements both before and after incubation with TTX. The ITO substrate proved biocompatible and non-toxic for N2a cells. TTX exposure caused 102% inhibition in EFP values at 300 nM, confirmed by sodium current inhibition of 93% at 300 nM and 22% at 1 nM in patch clamp studies (IC_50_ = 6.7 nM). EIS measurements indicated concentration-dependent impedance changes in the range of 6–300 nM. This research aims to provide a proof-of-concept for integration of electrophysiological and electrochemical approaches to simplify toxin detection systems.

## 1. Introduction

Tetrodotoxin (TTX), a thermostable potent neurotoxin that is found in species like *Lagocephalus sceleratus* (belonging to the Tetraodontidae family) and *Charonia lampas lampas* (shellfish), is known to cause food poisoning upon ingestion, resulting in paralysis and death in the most severe cases [1,2]. TTX affects the neuronal cells and central nervous system by inhibiting voltage-gated sodium channels (VGSC or Na_v_), which are essential for neuronal electrical connectivity. Toxicity studies are predominantly carried out using neuronal cell-based assays (CBAs), which take around 48–72 h to perform [3]. Other tests involve lab-based equipment like high-performance liquid chromatography (HPLC), which is used to detect the structure and quantity of toxin rather than the toxicity [4]. Recently, an automated patch clamp (APC) was used to detect and analyze the effect of toxin in real time [5]. But these methods are time-consuming, require expensive lab-based equipment, highly trained personnel, and maintenance. Currently, immuno-based assays [6], such as enzyme-linked immunosorbent assay (ELISA) [7,8,9,10], lateral flow assay (LFA) [11,12,13], and immunosensors [14,15,16,17,18], are widely used for marine toxin detection due to high specificity and low-cost, on-site, rapid detection. They generally use monoclonal antibodies to bind the toxin and are detected by a reporter in competitive or sandwich formats [19]. Commercial ELISA kits are also available for TTX detection [20,21,22]. However, they sometimes give rise to false positives. The production of antibody, toxin, and reporter conjugates is time-consuming, costly, and unable to differentiate between a toxin and its analogues.

Since TTX exhibits its toxicity by extracellular blockade of the channel pore of voltage-gated sodium channels (Na_v_), it might be advantageous to monitor the action potential of neuronal cells for early detection of the toxin. The most common methods employed for measuring neuronal activity are extracellular field potentials (EFP) and whole-cell manual patch clamp. Whole-cell patch clamp recordings can measure action potential and isolate currents from individual gating channels (e.g., Na_v_ or voltage-gated potassium channels (K_v_)). Upon stimulation, ionic fluxes can be measured as inward and outward currents that reflect the passage of specific ions through molecular targets, such as voltage-gated channels. Homeostasis requires the cell to transport ions out of the cell through passive transport, closing the current loop [23]. For EFP studies, the difference in the extracellular potential collectively from all cells in the electrode vicinity is recorded. In the presence of TTX, sodium intake is suppressed, which modulates the EFP and can be detected by the electrode. Therefore, the electrode characteristics, such as the material and its conductivity, are critically important for measuring the field potential accurately.

Indium tin oxide (ITO) is a commercially dominant transparent conductive oxide (TCO) used for glass and polyethylene terephthalate (PET) film substrates, combining high visible light transmission (>85%) with low electrical resistance [24]. ITO-coated substrates can be used to culture cells and evaluate cellular phenotypes and their conductive properties for electrical stimulation, providing a platform for simultaneous electrochemical and electrophysiological studies. Some studies were conducted using chromaffin cells plated on polypyrrole-modified ITO, which facilitated the detection of catecholamine secretion [25,26]. The studies include chronoamperometric detection of catecholamine release in response to high K^+^ exposure. However, it involves Ca^2+^ influx for exocytosis, facilitating electron transfer from cells to the electrode. Extracellular calcium levels affect sodium channel expression in numerous ways [27,28,29], one of which is reducing the excitability of voltage-gated sodium channels (VGSCs) [28]. Using calcium-free solutions in TTX studies maintains consistent sodium channel numbers, ensuring more reliable results. Using polymer-modified ITO multi-electrode arrays (MEAs) as a substrate, few researchers have used electrophysiological techniques for cell viability studies on primary hippocampal neurons and cardiomyocytes [30,31]. Many other biocompatibility in vitro studies have been conducted on cells grown on ITO films using electrochemical impedance spectroscopy (EIS) [32,33].

EIS is a non-invasive and label-free cell-based biosensor technology that has been used successfully for monitoring cell viability, growth, proliferation, and migration. However, with EIS, toxicity studies that rely on the mechanism of action of toxin on cells are less established. TTX and saxitoxin (STX) toxicity studies have been performed on cardiomyocytes with advanced EIS equipment, called the real-time cell analysis (RTCA) cardio system, which can record changes in impedance with rhythmic beats of cells in real time in a 96-well plate [34]. This system is costly for detection purposes and limited to lab-based detection.

The electrochemical investigation of cells grown on ITO electrodes relies on electron transfer mechanisms between the electrode and cells. Typically, this can be realized in two ways: direct electron transfer involving direct electrical contact from cells to the electrode or mediated electron transfer via a diffusion mediator or redox polymer layer attached to the electrode surface [35]. One such mediator, an Osmium complex modified redox polymer (poly(1-vinylimidazole-co-allylamine)-[Os(2,2′-bipyridine)_2_Cl]Cl), was used for electron transduction from microalgae [36]. However, microalgae respond differently to TTX, but still, the use of this redox polymer could provide a possibility to interact with neuronal cells.

On the other hand, Neuro-2a (N2a), murine neuroblastoma cells, are extensively used as a standard for assessment of marine toxins in CBAs [3,37]. Recently, cell viability studies have been conducted on N2a cells grown on ITO MEAs using a resonance-like impedance technique [38]. The use of a transparent electrochemically active substrate (such as ITO) for in vitro N2a cell culture should allow the electrochemical interrogation of a cell population (with techniques such as EIS or voltammetry) in parallel or simultaneously with EFP or patch clamp. The correlation of observations could support the use of such electrochemical methods for TTX detection.

Hence, in this work, N2a cells were used for the detection of TTX in real time using electrophysiological and electrochemical measurements. EFP studies have been conducted with N2a cells grown on ITO-coated glass (referred to as N2a/ITO) when evoked by potassium chloride (KCl) in the absence and presence of TTX. A corroborated whole-cell patch clamp study on N2a/ITO was used to develop a dose-dependent response curve for TTX. Parallel electrochemical studies have also been conducted on N2a/ITO, and an attempt was made to observe electrochemical signals from these cells by growing them on P-Os-modified ITO (referred to as poly-ITO and N2a/poly-ITO) through cyclic voltammetry (CV) and EIS.

## 2. Results

### 2.1. Confluency and Viability Studies

**Figure 1:**
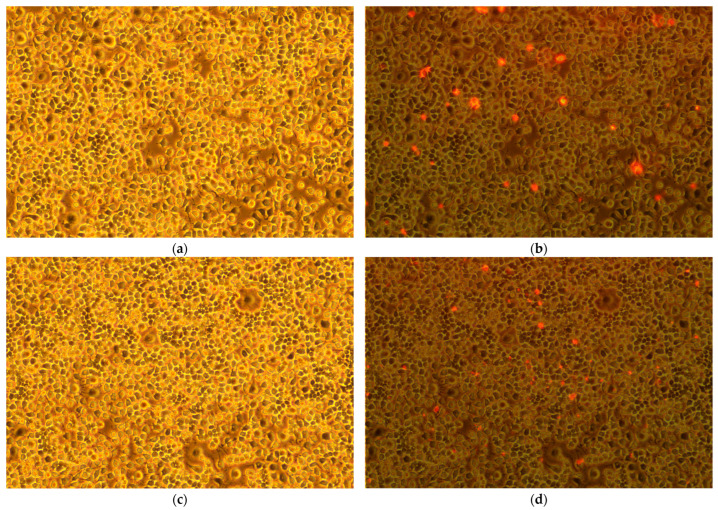
Bright field microscopy images: (**a**) Neuro-2a (N2a) cells on indium tin oxide (ITO) electrodes and (**b**) superimposed fluorescent image of dead cells. (**c**) N2a cells on plate and (**d**) superimposed fluorescent image of dead cells. All images were obtained at a magnification of 100×. Cells were dyed with propidium iodide for fluorescence image capturing.

N2a cells were grown on ITO-coated glass and plate for a 2-day period before testing microscopy images of N2a/ITO glass electrodes and N2a/plate, respectively. On day 3, on reaching confluency, microscopic and viability studies were performed (Figure 1). N2a cells reached 100% confluency covering the area shown, and no difference in cell viability and growth was detected using fluorescent labelling of dead cells, as shown in Figure 1b,d.

### 2.2. Extracellular Field Potential (EFP) Recordings on ITO

**Figure 2:**
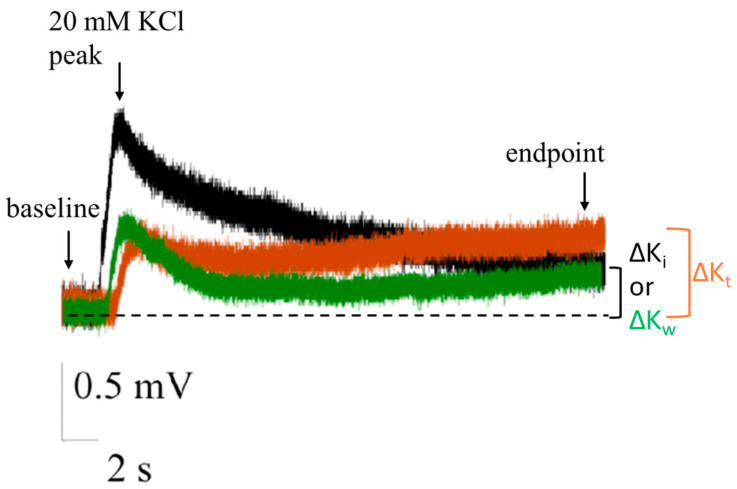
Extracellular field potential (EFP) recordings from N2a cells, plated on ITO with recordings for 30 s when 20 mM KCl is added, before tetrodotoxin (TTX) (black), after 5 min of incubation with TTX (orange), and after washout (green), respectively. Horizontal scale bar, 2 s and vertical scale bar 0.5 mV. Dotted line represents the baseline level. Note that the signals have been inverted.

Figure 2 presents EFP recordings of N2a cells cultured on indium tin oxide (ITO) substrates. The recordings were obtained during the following experimental sequence: stimulation with 20 mM KCl each time followed by washout, (1) prior to tetrodotoxin (TTX) application (black), (2) following a 5 min incubation with TTX (orange), and (3) post-washout (green). The potential difference between the baseline (initial point) and the peak voltage was indicative of the chemical stimulus, whereas the potential difference between the endpoint and the baseline provided insights into the blocking activity of TTX. An increase in peak potential was observed following KCl stimulation after TTX addition, with negligible or no potential drop at the end of the 30 s recording compared to KCl stimulation before TTX addition. The KCl-induced activity partially resumed after washout (replenishment with Locke-4-(2-Hydroxyethyl)-1-piperazineethanesulfonic acid (HEPES) buffer solution) in ITO samples.

**Figure 3:**
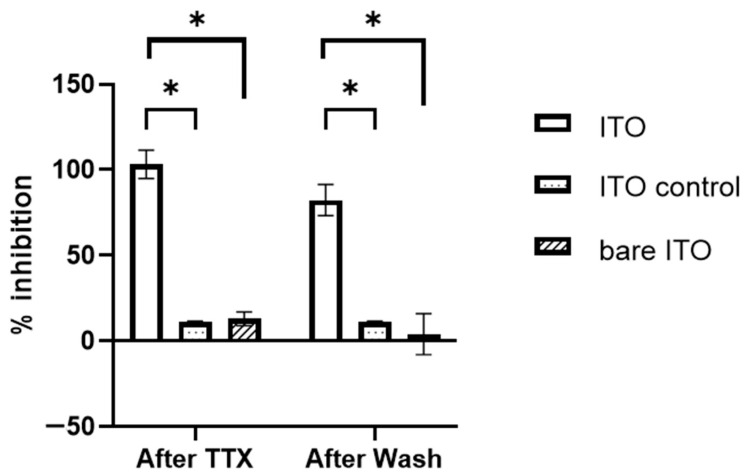
% inhibition from initial KCl action before TTX, of N2a cells on ITO tested with TTX (white), N2a cells on ITO tested without TTX, and bare ITO (without cells) tested with TTX (shaded). For the control experiments, TTX-free buffer was used instead of TTX. Values are expressed as Mean ± SEM (n ≥ 3). * (*p* < 0.05) (multiple *t*-test).

In our investigation of neuronal activity, we observed significant changes in EFP differences when N2a cells were subjected to various stimuli on ITO substrates, expressed as % inhibition (please see Section 5.7 for % inhibition calculation), and values are illustrated in Figure 3. The application of KCl following a 5 min incubation with TTX resulted in 103 (±8.3 SEM, n = 10) % inhibition for ITO. This shows desensitization of voltage-gated sodium channels of the neurons to stimuli after TTX. After TTX washout, we observed a differential recovery of the KCl-evoked signal between the two substrates. The ITO samples demonstrated a 10% recovery of the original signal.

To confirm our results and control for potential confounding factors, we conducted several additional tests. Control experiments were performed by adding TTX-free buffer instead of TTX, following the same protocol. This step was crucial to distinguish between the specific effects of TTX and any non-specific buffer effects that might influence the observed EFP differences. Furthermore, to eliminate the possibility of substrate-induced artifacts, we carried out the same experimental protocol on bare ITO substrates without cells (supporting Figure S1). This control measure allowed us to isolate the cellular response from any potential artifacts generated by the ITO surface itself.

### 2.3. Patch Clamp Recordings on ITO

**Figure 4:**
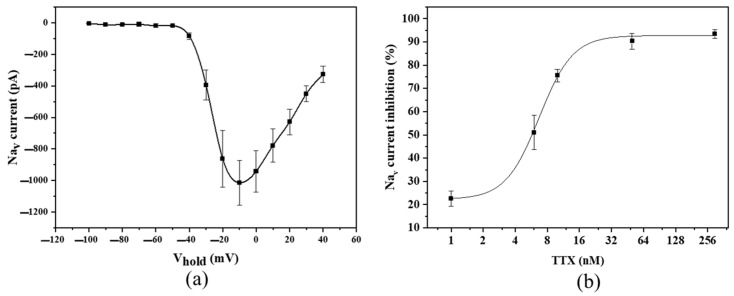
Effects of TTX on N2a sodium currents, (**a**) Na_v_ currents recorded from N2a cells (n = 3) on ITO-coated glass prior to TTX exposure, and (**b**) dose–response curve for TTX showing the blocking effect of this toxin on Na_v_ currents in N2a cells. The curve has been fitted with a logistic model with parameters shown in the inset of the graph. (n ≥ 3). Values are expressed as Mean ± SD.

To establish a standardized dose-dependent response curve for TTX using patch clamp for the N2a/ITO, the electrophysiological activity of these cells was initially assessed prior to the toxin exposure (Figure 4a). The undifferentiated N2a cells without any blocking pretreatment measures were subjected to voltage clamping in whole-cell configuration, and in this work, the peak current achieved was −1013 pA at −10 mV, +60 mV from the resting potential, which is −70 mV.

Furthermore, the blocking activity of TTX was assessed. The N2a cells were subjected to toxin concentrations of 1, 6, 10, 50, and 300 nM gradually. The recordings were continuously monitored to observe the stabilization of inhibition at every dosage and to detect loss of cell (leakage currents). Almost complete inhibition of Na_v_ currents was observed at 300 nM concentrations (93% approx.), whereas 20% of signal inhibition was observed at the initial 1 nM concentration. The dose–response curve for TTX was developed (Figure 4b) and fitted with a four-parameter logistic curve model with an R^2^ value > 0.9999. From the fit, an IC_50_ value of 6.7 nM was calculated.

### 2.4. Electrochemical Experiments on ITO

Electrochemical measurements were conducted alongside patch clamp experiments on N2a cells cultured on ITO (N2a/ITO) and on poly-ITO substrates (N2a/poly-ITO). Initially, CV was employed to investigate the electrochemical behavior of the cells and the effect of TTX at various concentrations, followed by EIS. CV of N2a/ITO electrodes revealed no specific voltage-dependent redox characteristics. In contrast, N2a/poly-ITO electrodes exhibited distinct redox activity, with anodic and cathodic peaks observed at 0.43 V and 0.39 V, respectively. The presence of cells on both ITO and poly-ITO surfaces resulted in a decreased capacitive current compared to cell-free electrodes. Therefore, although cyclic voltammetry provides an easy method to verify cell immobilization in situ (by observing the modulation of capacitive currents), it did not offer insights or differentiating features that correlate to the change in cell properties upon exposure to TTX. To address this limitation and gain a deeper understanding of the impedance characteristics and their modulation by TTX, EIS measurements were conducted.

**Figure 5:**
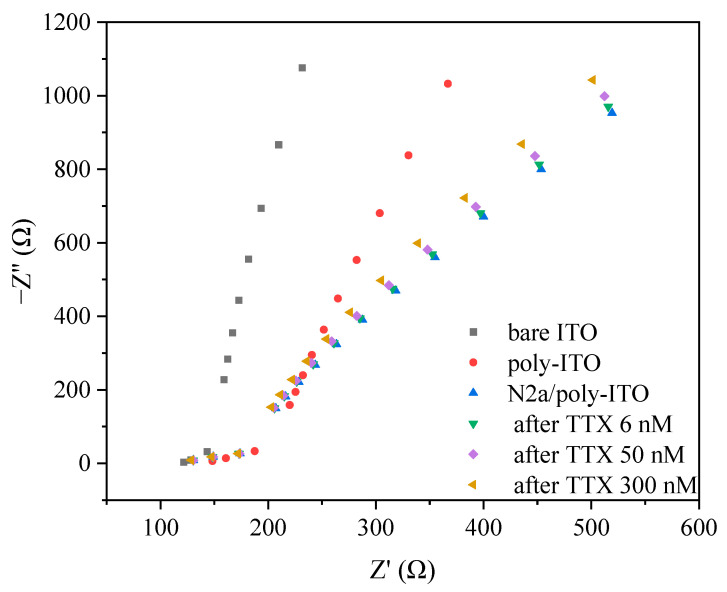
Electrochemical impedance spectroscopy (EIS) measurements of bare ITO (square), poly-ITO (circle), N2a/poly-ITO (upward triangle), after 5 min. incubation with TTX 6 nM (downward triangle), 50 nM (diamond), 300 nM (tilted triangle). Experimental conditions detailed in Section 5.6.

**Figure 6:**
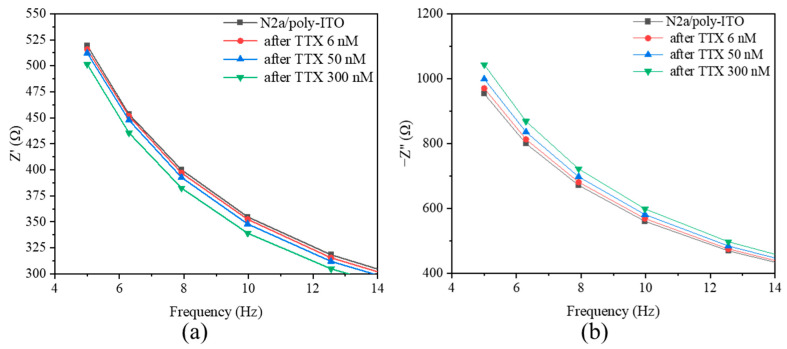
Frequency versus (**a**) real (Z’) and (**b**) imaginary(−Z’’) impedance from 5 to 13 Hz, representation: N2a/polyITO (black square), after 5 min. incubation with TTX −6 nM (red circle), 50 nM (blue upward triangle), and 300 nM (green downward triangle).

EIS measurements were conducted on bare ITO, poly-ITO, and N2a/poly-ITO substrates in the absence and presence of TTX at concentrations of 6, 50, and 300 nM (Figure 5). The Nyquist plots show rightward shift of data points for poly-ITO compared to bare ITO, indicating the presence of the polymer, and for N2a/poly-ITO compared to poly-ITO, indicating the presence of cells. Concurrently, a subtle change in real (Z’) and imaginary impedance (−Z”) was observed, with points shifting slightly leftward in response to increasing TTX concentrations. Whereas N2a/ITO, when tested with TTX concentrations for impedance changes, no such correlation was found. Thus, these impedance changes in N2a/poly-ITO, while subtle, are statistically significant and may reflect the change in the system’s electrochemical properties in response to the neurotoxin. Magnified frequency vs. impedance graphs in Figure 6a,b, show a TTX concentration-dependent trend of the impedance change as a function of frequency with increasing TTX concentrations in comparison to N2a/poly-ITO (without TTX), especially in the region of 5 to 13 Hz.

**Figure 7:**
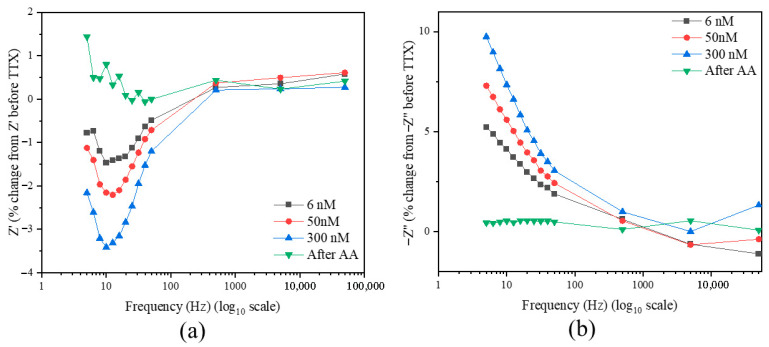
Modified Bode-type plots with % change, (**a**) real (Z’) and (**b**) imaginary(−Z’’) impedance with respect to frequency from 50k Hz to 5 Hz plotted on log10 scale, representation: after 5 min. incubation with TTX; 6 nM (black square), 50 nM (red diamond), 300 nM (blue upward triangle), and after equivalent amount (for 300 nM TTX) of TTX-free acetic acid (after AA) (green downward triangle).

Modified Bode-type plots (Figure 7) provide a more detailed analysis of these impedance changes. It presents the percentage change in Z’ and −Z’’ values at 6 nM, 50 nM, and 300 nM TTX concentrations, as well as after acetic acid addition (control), relative to initial values before TTX exposure. Across the frequency range from 50 kHz to 5 Hz, the percentage change in Z’ gradually decreased, becoming more negative, especially at lower frequencies (Figure 7a). Conversely, the percentage change in −Z’’ increased progressively, contrasting with the values observed after acetic acid addition (Figure 7b). The concentration-dependent effect of TTX is evident, with larger differences observed at higher concentrations and lower frequencies (100 to 5 Hz). Notably, no significant change was detected following the addition of acetic acid, serving as a control.

## 3. Discussion

The purpose of this study is to evaluate ITO-coated substrates as a viable alternative to conventional inert substrates for in vitro cell culture applications. This study aims to explore the potential benefits and limitations of ITO-coated surfaces in supporting cell growth, adhesion, and proliferation, while also investigating their compatibility with N2a cells. Assessment of N2a/ITO substrates demonstrates successful cell adhesion and proliferation, with confluency analysis indicating robust cell coverage, comparable to that on an inert substrate. Viability tests using propidium iodide staining reveal excellent cell health, with less than 3% cell death observed.

This study also aims to provide a proof-of-concept integration of electrophysiological and electrochemical approaches using Neuro-2a (N2a) cells on indium tin oxide (ITO) substrates. Our work demonstrates the feasibility and biocompatibility of ITO (without full validation) for its integration into electrophysiological and electrochemical impedance cell-based toxicity studies. Thus, N2a cells cultured on indium tin oxide (ITO) substrates exhibited a distinct electrophysiological response when stimulated with KCl, characterized by an initial increase in EFP followed by a decrease over 30 s, while bare ITO showed minimal to no change, confirming the response is cell-mediated. Considering the inverted signal, KCl-induced depolarization opens the VGSCs, allowing Na^+^ influx (into the cells) that causes the initial potential peak, followed by recovery to homeostasis through K^+^ efflux, consistent with the Hodgkin–Huxley kinetic models stated in [39] and EFP recorded in the absence and presence of TTX in other works [40,41]. Thus, the EFP response is not directly due to the stimulus itself but rather the ion dynamics it triggers. The lack of Ca^2+^ in the solution prolongs the recovery of the signal back to the baseline as it affects Ca^2+^-dependent K^+^ channels. Thus, there is a subsequent delay in achieving homeostasis (up to 30 s).

The presence of TTX blocks the VGSCs (no Na^+^ influx into the cells) and causes a rise in the extracellular ionic concentrations at the endpoint (at 30 s). This rise, in comparison to EFP results before TTX, creates a difference, which is reported as the TTX-related signal. After washout, a similar reduced peak but subsequent drop in potential to that of the initial KCl action was observed, indicating some of the channels have been unblocked by the washout. A 100% recovery was not observed for the ITO cells, which may be attributed to the remnant TTX in solution left out between the ITO and culture dish due to capillary action when the solution is suctioned off by vacuum for wash-off. Moreover, although TTX is considered a reversible inhibitor/blocker, it tends to stick to the TTX-sensitive Na^+^ gating channels (due to low disassociation constant (Kd ~ 1–5 nM) [42,43]. However, in a sensor application, the washout reading would not be necessary (in the current study, it is used as a control to study the stability of the system). Moreover, the 103 (±8) % inhibition at 300 nM TTX of EFP results corresponds to the 93 (±1.85) % inhibition at the same concentration observed in the patch clamp studies.

Traditionally, when CBAs are performed with N2a cells for assessing toxicity of TTX, they are initially pretreated with ouabain, a Na^+^/K^+^ ATPase inhibitor, and veratridine, a Na^+^ channel activator. The combined effect of these two causes continuous depolarization of the N2a cells, ultimately leading to cell death [3]. However, the composition is adjusted to cause cell mortality to 80%, and TTX is added to counteract them in a dose-dependent manner. The cell viability is monitored over 72 h. This entire process is labor-intensive and time-consuming. In contrast, EFP and EIS studies provide toxin detection in real-time, without the use of ouabain and veratridine.

In our study, we employed a single concentration of 300 nM tetrodotoxin (TTX) for extracellular field potential (EFP) recordings on indium tin oxide (ITO) substrates. This was selected as a threshold to achieve near-complete inhibition of voltage-gated sodium channels, based on prior toxicity studies with Neuro 2a cell-based assays (~96 ng/mL), which are used to confirm maximum blockade and differentiate from partial effects [3]. This approach, while not capturing the full dose–response profile, serves as an effective initial screening method to detect TTX at a concentration sufficient to achieve complete inhibition of voltage-gated sodium channels, as observed in our experiments. This aligns with practical screening objectives, where the aim is to rapidly confirm the presence of TTX above a critical threshold rather than to quantify its concentration across a range. The method’s sensitivity and speed are vital, given that TTX poisoning can occur from exposures ranging from 4 to 42 μg/kg body weight [44], a range reflecting observed human poisoning cases. For comparison, a portable high-throughput biosensor based on cardiomyocytes can detect TTX at 0.30 ng/mL (equivalent to 0.94 nM) within 10 min, highlighting the feasibility of detecting TTX at far lower concentrations than our 300 nM screening threshold [16]. In the context of food safety, the European Food Safety Authority (EFSA) CONTAM Panel has established that a TTX concentration below 44 μg/kg (138 nM assuming extraction of 100 g of sample in 100 mL) in shellfish meat, based on a 400 g portion, is unlikely to cause adverse effects in humans, which is below our screening concentration but within the detectable range of sensitive electrophysiological methods. Recognizing the limitations of a single-concentration approach, we complemented our EFP recordings with whole-cell patch-clamp studies on Neuro-2a cells grown on ITO substrates, developing a calibration curve to characterize the dose–response relationship. In future work, the dose–response curve for TTX detection will be established through EFP experiments to validate the sensitivity and dynamic range of the ITO-based cell detection system. Moreover, the patch clamp studies yielded an IC_50_ value of 6.7 nM for TTX, closely aligning with the 6.4 nM reported in recent literature [5], confirming the compound’s high potency in our system. This IC_50_ indicates that our method is sensitive enough to detect TTX at concentrations well below both the 300 nM screening level and the regulatory threshold, enhancing its applicability for TTX monitoring in marine products. The linear detection range of the ITO-based patch clamp system is around 2–32 nM (0.638–10.21 ng/mL), which is limited when compared to N2a-based assay and detection systems. Therefore, to address the need for a comparative evaluation of TTX detection methods, Table 1 is provided, with a detailed comparison of the proposed ITO-based method against established techniques with limit of detection (LOD), detection range (limit of quantification to maximum), and advantages and disadvantages, highlighting its competitive sensitivity and rapid detection capabilities within the context of regulatory requirements and alternative approaches. By integrating rapid screening with detailed electrophysiological analysis, our approach balances practicality with the need for comprehensive toxicological insights, despite challenges such as limited availability of certified TTX standards.

The EIS measurements revealed statistically significant changes in impedance upon TTX exposure for cultures of N2a cells on ITO substrates (Figure 6 and Figure 7). These changes are more important in the low-frequency region. The observed increase in real impedance (Z’) with decreasing frequency for poly-ITO and N2a/poly-ITO substrates aligns with previous studies on cell-substrate interactions [33,50]. This increase can be attributed to the formation of a more resistive layer due to cell adhesion and the presence of the polymer coating. As in the previous works mentioned, this data can be used to ascertain cell coverage. The TTX-concentration-dependent shifts in impedance components upon TTX addition is indicative of possible modulation of the apparent capacitance of the polymer/electrode/electrolyte interface, possibly due to counterion penetration and/or mobility or even of the effect of the toxin on cellular electrical properties that can be captured by this interface, since no similar effect was observed on N2a/ITO electrodes (that were not modified by P-Os). Modeling from first principles of the EIS response of redox polymer interfaces [51,52] strongly suggests that such apparent capacitance modulation is due to counterion mobility and permeability in the low frequency region. The % impedance change as a function of frequency with increasing TTX concentration may even indicate alterations in the cell membrane properties and ion channel function, consistent with TTX’s known mechanism of action [53]. The lack of significant changes after acetic acid addition (control) further confirms the TTX effects. This impedance-based approach offers a label-free, real-time method for detecting TTX-induced cellular changes, complementing traditional electrophysiological techniques on ITO. The sensitivity of this method, capable of detecting changes at concentrations as low as 6 nM (<IC_50_), demonstrates its potential for early detection of TTX contamination in food safety applications, aligning with recent advancements in portable sensing systems for marine toxin detection [46]. Moreover, since this is a cell-based detection system, it can be used for measuring the performance with other marine toxins like brevetoxin, saxitoxin, and domoic acid, which arise from harmful algal blooms (HABs) and leach into the sea upon cell lysis, without any need for pretreatment or studying matrix effects of fish or shellfish [54].

## 4. Conclusions

This study demonstrates that indium tin oxide (ITO) electrodes provide an effective and biocompatible platform for real-time detection of tetrodotoxin (TTX). Experiments with Neuro-2a cells confirmed that TTX exposure leads to an increase in extracellular field potential (EFP) and significant inhibition of sodium currents in patch clamp, with an IC_50_ of 6.7 nM. Additionally, EIS studies revealed concentration-dependent changes in response to TTX concentrations from 6 to 300 nM, reinforcing the feasibility of ITO for biosensor applications.

The use of ITO enables the integration of electrophysiological and electrochemical measurements into a single system, reducing the complexity and costs associated with traditional methods. This approach provides a proof-of-concept for developing portable and cost-effective sensors for marine toxin detection, with potential applications in food safety and environmental monitoring. At the same time, this study reveals the need to further characterize the resulting platform for its analytical merits, with an extensive selectivity study, developing a dose–response curve and validation with real samples. It would also be of interest to study the mechanism that gives rise to the impedance modulation. Such knowledge could provide the basis for development of a multi-toxin detection platform if the signal modulation can be differentiated in the time or frequency domain.

## 5. Materials and Methods

### 5.1. Cells

N2a cells were purchased from ATCC, LGC standards (Manassas, VA, USA). N2a cells were cultured in Rosewell Park Memorial Institute (RPMI) 1640 medium containing L-glutamine and phenol red. The medium was supplemented with 10% fetal bovine serum (FBS), penicillin (0.01 mg/mL), streptomycin (10 U/mL), and 1 mM sodium pyruvate. The cells between passages 248 and 257 were treated under sterile conditions in a vertical laminar flow cabinet and maintained in an incubator at 37 °C with 5% CO_2_ humid atmosphere.

### 5.2. Reagents

Tetrodotoxin (TTX) was purchased from Tocris Bioscience (Bristol, UK) and prepared as a stock solution at 1 mg/mL in 1% acetic acid. Dilutions to 30 µM and 300 nM were prepared and stored at −20 °C until use. RPMI 1640 medium, sodium pyruvate, Foetal Bovine Serum (FBS), Phosphate Buffer Saline (PBS), Trypsin-Ethylenediaminetetraacetic acid (EDTA) enzyme, NaCl, KCl, 4-(2-Hydroxyethyl)-1-piperazineethanesulfonic acid (HEPES), D-Glucose, MgCl_2_, and Indium Tin Oxide (ITO)-coated glass slides of 25mm × 25 mm squares were purchased from Merck KGaA (Darmstadt, Germany) and sectioned to desired size using a CO_2_ laser cutter. Poly(1-vinylimidazole)-[Os(4-4′-dimethoxy-2,2′-bipyridine)_2_Cl]Cl) (P-Os) was obtained as a gift. The synthesis method is as described in [55].

### 5.3. Cell Plating and Viability

ITO-coated glass (sectioned into 6.25 mm × 12.5 mm rectangles) was used for culturing the cells. Prior to culturing, the ITO electrodes were first rinsed in soap, deionized water, and ethanol and dried for 30 min under sterile conditions. The cells were cultured onto ITO-coated glass (N2a/ITO) by placing them on the 30 mm polystyrene culture dish two days before the experiment to reach confluency at the time of experiment. In parallel, the cells were also grown directly on a 30 mm polystyrene culture dish (N2a/plate) to confluency for the comparison. Cell viability was tested using propidium iodide dye (5 µL/mL of medium), incubated for 10 min at 37 °C, and checked with a fluorescence microscope under green wavelength. Before the experiments, the medium was replaced with PBS to remove dead cells and subsequently with Locke-HEPES buffer (140 mM NaCl, 5 mM KCl, 10 mM HEPES, 10 mM Glucose, and 1.2 mM MgCl_2_, pH 7).

### 5.4. Extracellular Field Potential (EFP) Recordings

**Scheme 1:**
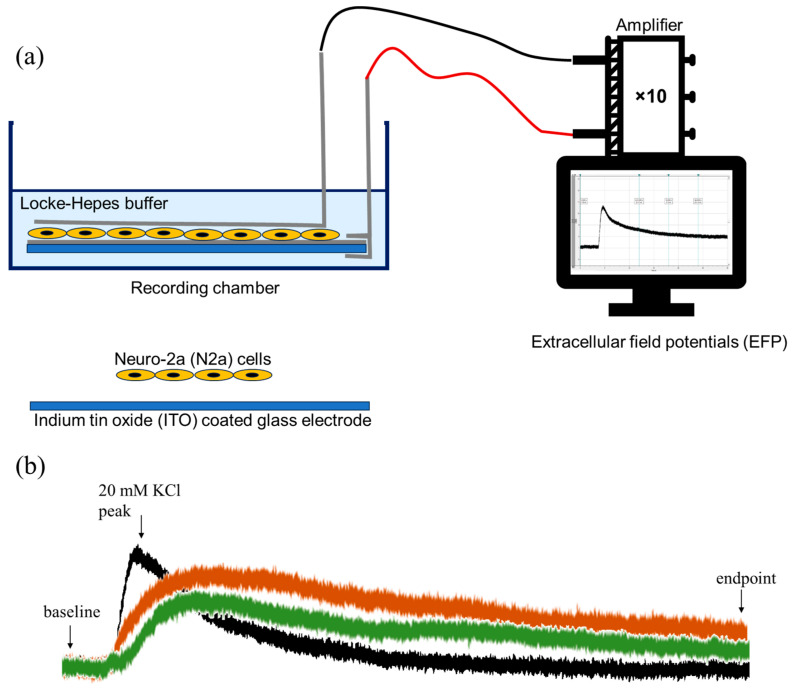
(**a**) A schematic of EFP experimental setup and (**b**) an illustration of measuring KCl-evoked EFP, before (black), after 5 min. incubation with TTX (red), and after washout (green). The variation in endpoint to baseline difference provides insights into the effect of TTX.

A protocol was developed to record the signals each time the cells were evoked with KCl before TTX, after TTX (300 nM final concentration in the bath) with 5 min incubation, and after washout. Recording electrodes were connected to an amplifier (Tecktronics, AMS02, Beaverton, OR, USA), and a distal Ag-AgCl electrode connected to the bath solution was used as reference (see Scheme 1a). The signals were digitized (DIGIDATA 1322A Interface, Axon Instruments Inc., Weatherford, TX, USA), stored, and treated. The software Axoscope 10.0 (Axon Instruments Inc.) was used for data acquisition and analysis. An illustration of how the extracellular field potentials are measured is shown in Scheme 1b. It should be noted that the signal has been inverted for calculation purposes. The field potential difference is measured from endpoint to baseline. The percentage variation between the field potential difference before and after addition of TTX is calculated.

### 5.5. Patch Clamp Experiments on ITO

All recordings were conducted in the whole-cell configuration using a manual patch clamp setup. As in EFP recordings, N2a/ITO were also used for the purpose of patching. In the whole-cell configuration, a specified cell is touched with a glass micropipette (D = 2–5 µm ~ adjusted to the cell size), and the cell is pulled into the micropipette through suction, creating a gigaseal (>1 GΩ). The capacitance was recorded as a measure of cell size. Voltage clamp experiments were performed with resting potential at −70 mV. To evoke Na_v_ currents, the potential was stepped from −100 to +40 mV, in 10 mV increments for 200 ms. An intracellular solution (50 mM CsCl, 60 mM CsF, 10 mM NaCl, 20 mM EGTA, 10 mM HEPES/CsOH pH 7.2) was loaded into the micropipette for patching the cell. An Ag/AgCl wire (D = 1.5 mm) was connected to a pipette holder, which was further mounted to the headstage. A second Ag/AgCl wire, which was also connected to the headstage, served as the reference electrode in the electrolyte bath. Both Ag/AgCl were chlorinated regularly with Clorox bleach around 10–15 min before the measurements. Recordings were averaged over 60 sweeps. Data were acquired and digitized via a Digidata 1322A in combination with an Axopatch 200B amplifier (Molecular Devices, Foster City, CA, USA) and Clampex 10.7 software (Molecular Devices, Foster City, CA, USA). Currents were sampled at 2k Hz and low-pass filtered at 1k Hz. Offline data analysis was conducted using Clampfit 10.7 software (Molecular Devices, Foster City, CA, USA).

### 5.6. Electrochemical Experiments

Parallel electrochemistry experiments were also performed along with patch clamp experiments. Apart from a patch clamp 2-electrode setup, an external Ag/AgCl reference electrode and a Pt wire as counter electrode were used for all measurements. Cyclic voltammograms (CV) and EIS measurements were recorded with a portable potentiostat (PalmSens 4), which was controlled, and data was analyzed using PSTrace 5.10 software. EIS measurements were performed with a frequency range of 50k Hz to 5 Hz with amplitude (E_ac_) of 0.01 V [56] and E_dc_ of 0.45 V (redox potential of P-Os polymer). For each experiment, a minimum of 3 electrodes were tested to assess reproducibility. All the experiments were conducted at room temperature, submerged in Locke-HEPES buffer. For poly-ITO, 2 µL of 1.7 mg/mL (P-Os) polymer solution was evenly spread onto the clean, sterile ITO electrode (0.78125 cm^2^), and cells were cultured as described previously in Section 5.3.

### 5.7. Statistical Analysis

The values of EFP experiments are expressed as “percentage of change from potential difference of initial KCl action (ΔK_i_) to potential difference of KCl action after TTX (ΔK_t_) or after washout (ΔK_w_)”

Where

ΔK_i_ or ΔK_t_ = [endpoint−baseline value] of the corresponding recording, then (1)

% inhibition = (ΔK_i_ − ΔK_t_) × 100/ΔK_i_ (2)

Data was analyzed using GraphPad Prism 10.4.1 (GraphPad Software LLC, Boston, MA, USA). Values are expressed as mean ± standard error of means (SEM)/standard deviation (SD), considering the 95% confidence interval. The statistical analyses were performed with stricter multiple unpaired *t*-tests with Welch correction (Holm–Sidak method) for statistical significance. Differences were considered significant at *p* < 0.05.

## Figures and Tables

**Table 1:** The table summarizes key TTX detection methods with advantages and disadvantages, including their limit of detection (LOD) and linear detection range, expressed in consistent units (in ng/mL unless otherwise stated, where 1 nM TTX ≈ 0.319 ng/mL based on molecular weight of 319.27 g/mol).

Method	Lowest LOD (ng/mL ^1^)	Linear Detection Range (ng/mL)	Advantages	Disadvantages and Requirements	Reference
Mouse Bioassay (MBA)	1.1–2.2 mg/g of mouse	NS ^2^	Assesses overall toxicity, no equipment needed	Ethical concerns; low specificity, time-consuming (24–48 h)	[45]
Neuro-2a (N2a) cell-based assay (CBA)	0.32–1.6	0.96–95.7	Measures functional toxicity and TEFs, sensitive, cost-effective	Requires cell culture and pretreatment (e.g., ouabain/veratridine), 24–72 h duration, labor-intensive	[3]
High-performance liquid chromatography coupled with tandem mass spectrometry (HPLC-MS/MS)	0.1–1	0.3–100	High accuracy, quantifies analogs, gold standard	Expensive equipment, sample preparation required, lab-based	[46]
Automated patch clamp (N2a cell-based)	0.32	15.95–95.7	Real-time, high throughput	Costly, requires skilled operators	[5]
Enzyme-linked immunosorbent assay (ELISA)	4.44	5–500	fast (1–2 h), portable, low cost	Development cost	[47]
Lateral flow assay (LFA) (immuno/aptamer-based)	0.2 (competitive), 0.3 (sandwich)	1.56–100	On-site, visual readout; quick (<15 min)	Semi-quantitative; lower sensitivity	[13,19]
Aptasensors	6 pg/mL	0.01–300	High specificity, stable aptamers	Development cost, limited commercial availability	[48]
Immunosensors	7 pg/mL	0.001–100	Ultra-high specificity	Development cost, limited commercial availability	[49]
Special cell-based EIS sensor, real-time cell analysis (RTCA)	89	0.001–100	Real-time, sensitive, portable	Potential fouling, requires real sample validation	[34]
N2a/ITO-based extracellular field potential (EFP)	95.8 ^3^	NA ^4^	Cost-effective; rapid (<10 min)	Non-specific; requires dose–response curve and real sample validation	This study
N2a/ITO-based patch clamp	0.32	0.63–10.21	Real-time	Expensive and requires skilled operators and real-sample validation	“
N2a/ITO-based, electrochemical impedance spectroscopy (EIS)	1.92	1.92–95.8	Cost-effective; rapid (<10 min)	Non-specific and requires real sample validation	“

^1^ unless stated otherwise, ^2^ NS—not stated, ^3^ preliminary, ^4^ NA—not assessed.

## Data Availability

The original contributions presented in this study are included in the article/supplementary material. Further inquiries can be directed to the corresponding author.

## Supplementary Materials

The following Supporting Information can be downloaded at: https://www.mdpi.com/article/10.3390/toxins17090462/s1, Figure S1: EFP recordings on bare ITO (without cells) (a) before TTX, (b) after TTX, and (c) after wash.

## Abbreviations

The following abbreviations are used in this manuscript:

APC	Automated patch clamp
CBA	Cell-based assay
EDTA	Ethylenediaminetetraacetic acid
EFP	Extracellular field potential
EFSA	European Food Safety Authority
EIS	Electrochemical Impedance Spectroscopy
ELISA	Enzyme-linked immunosorbent assay
HABs	Harmful algal blooms
HEPES	4-(2-Hydroxyethyl)-1-piperazineethanesulfonic acid
HPLC	High-performance liquid chromatography
IC_50_	Half-maximal inhibitory concentration
ITO	Indium tin oxide
LFA	Lateral flow assay
LOD	Limit of detection
MEA	Multi-electrode array
N2a	Neuro-2a cell lines
PET	Polyethylene terephthalate
P-Os redox polymer	Poly(1-vinylimidazole-co-allylamine)-[Os(2,2′-bipyridine)_2_Cl]Cl
RPMI	Rosewell Park Memorial Institute
RTCA	Real-time cell analysis
SD	Standard deviation
SEM	Standard error of means
STX	Saxitoxin
TTX	Tetrodotoxin
TCO	Transparent conductive oxide
VGSC	Voltage-gated sodium channels
