# Supplementary material for: Cost-Effective and Rapid Detection of Tetrodotoxin Using Indium Tin Oxide Electrodes via In Vitro Electrophysiology and Electrochemistry
Source: Toxins (Basel). 2025 Sep 13;17(9):462. doi: 10.3390/toxins17090462 (PMC12474310; doi:10.3390/toxins17090462)
Supplement: Supplementary file 1 [file toxins-17-00462-s001.zip › toxins-3807378-supplementary.pdf]

Cost effective and rapid detection of tetrodotoxin using indium tin oxide electrodes via in-vitro electrophysiology and electrochemistry

Naga Adithya Chandra Pandurangi, Manel M. Santafe, Angels Tudo, Nagihan Ozsoy, Fransesc X. Sureda, Mark L. Dallas and Ioanis Katakis

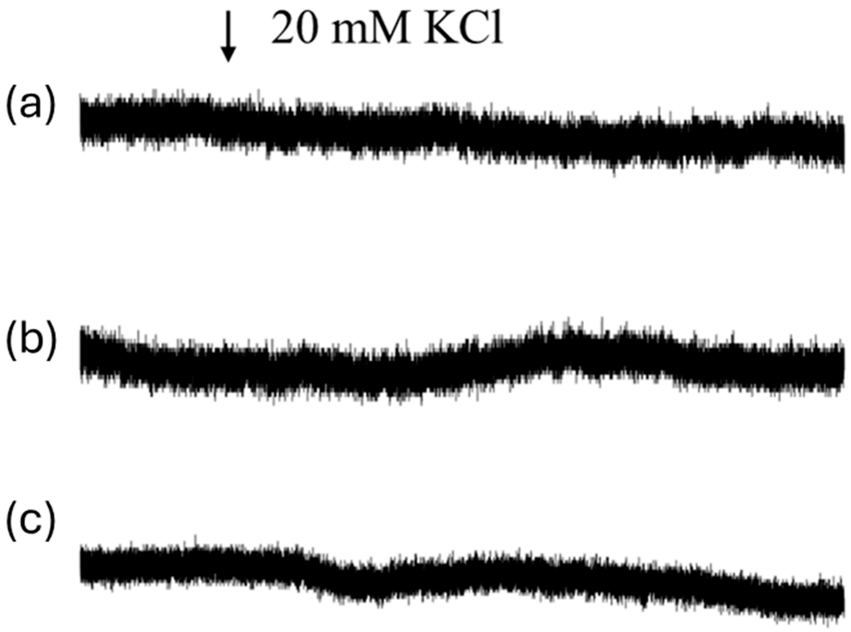

Figure S1. EFP recordings on bare ITO (without cells) (a) before TTX, (b) after TTX and (c) after wash.
